# Free Triiodothyronine Concentrations Are Inversely Associated with Microalbuminuria

**DOI:** 10.1155/2014/959781

**Published:** 2014-11-16

**Authors:** Yulin Zhou, Lei Ye, Tiange Wang, Jie Hong, Yufang Bi, Jie Zhang, Baihui Xu, Jichao Sun, Xiaolin Huang, Min Xu

**Affiliations:** ^1^Shanghai Clinical Center for Endocrine and Metabolic Diseases, Shanghai Institute of Endocrine and Metabolic Diseases, Department of Endocrinology and Metabolism, Rui-Jin Hospital, Shanghai Jiao-Tong University School of Medicine, 197 Rui-Jin 2nd Road, Shanghai 200025, China; ^2^Key Laboratory for Endocrine and Metabolic Diseases of Ministry of Health, Rui-Jin Hospital, Shanghai Jiao-Tong University School of Medicine, E-Institute of Shanghai Universities, Shanghai 200025, China

## Abstract

Thyroid function and microalbuminuria are both associated with vascular disease and endothelial damage. However, whether thyroid function is associated with microalbuminuria is not well established. The objective was to explore the relationship between thyroid hormones and microalbuminuria in Chinese population. A community-based cross-sectional study was performed among 3,346 Chinese adults (aged ≥ 40 years). Serum free triiodothyronine (FT3), free thyroxine (FT4), and TSH (thyroid stimulating hormone) were determined by chemiluminescent microparticle immunoassay. A single-void first morning urine sample was obtained for urinary albumin-creatinine ratio measurement. The prevalence of microalbuminuria decreased according to FT3 quartiles (13.2, 9.5, 8.6, and 8.2%, *P* for trend = 0.0005). A fully adjusted logistic regression analysis showed that high FT3 levels were associated with low prevalent microalbuminuria. The adjusted odds ratios for microalbuminuria were 0.61 (95% CI, 0.43–0.87, *P* = 0.007) when comparing the highest with the lowest quartile of FT3. The exclusion of participants with abnormal FT3 did not appreciably change the results (OR = 0.69, 95% CI, 0.49–0.98, *P* = 0.02). We concluded that serum FT3 levels, even within the normal range, were inversely associated with microalbuminuria in middle-aged and elderly Chinese adults. FT3 concentrations might play a role in the pathogenesis of microalbuminuria.

## 1. Introduction

Microalbuminuria was first reported in diabetic patients by Viberti et al. in 1982 [1]. It was defined as the urine albumin-to-creatinine ratio (UACR) of 30 to 300 mg/g and originally used to predict chronic kidney disease and diabetic nephropathy [2]. Previous studies have confirmed that there is an increased risk of cardiovascular disease and death related to microalbuminuria [3]. The association between microalbuminuria, progressive atherosclerotic vascular disease, and renal damage has been well established in patients with systemic diseases [4]. Microalbuminuria is extensively believed to reflect a generalized impairment of the endothelium [5] which is an important determinant of vascular disease.

Thyroid hormones are also found to be associated with endothelial damage and cardiovascular disease [6–9]. Previous studies have shown that thyroid hormones, via dilation of blood vessel, production of vasodilator molecules, inhibition of angiotensin II receptor, and its signal transduction, regulate endothelial function and vascular homeostasis [7, 8]. Moreover, in experimental models, triiodothyronine (T3) had important effects on the vascular system, such as inducing relaxation of vascular smooth muscle cells through a direct or indirect effect [8, 9]. The epidemiology study of microalbuminuria also revealed a close association between systemic endothelial dysfunction and vascular disease [10]. However, evidence for the association between thyroid hormones and microalbuminuria in a community-based study, if any, is scarce. Just recently, Mervat and colleagues reported that subclinical hypothyroidism is independently associated with microalbuminuria in 147 prediabetic adults [11]. But the sample size was relatively small and it was not a community-based study.

In this large community-based cross-sectional study, we aimed to investigate the relationship between thyroid hormones and microalbuminuria in middle-aged and elderly Chinese population.

## 2. Subjects and Methods

### 2.1. Study Population and Design

A cross-sectional survey was performed during 2008-2009 in a population from Songnan community located in Baoshan District, Shanghai. The selection criteria and design of the study have been described previously [12]. Briefly, in phase 1 (June and July 2008), we conducted the investigation on residents aged 40 years or older. A total of 10,185 participants were included in the screening examination. All participants received a fasting blood test. Based on the results of fasting glucose level and history of diabetes, participants were categorized into three groups: normal glucose regulation (NGR), defined as a fasting plasma glucose level less than 5.6 mmol/L (<101 mg/dL) and no history of diabetes; impaired glucose regulation (IGR), defined as a fasting plasma glucose level of 5.6 to less than 7.0 mmol/L (101 to <126 mg/dL) and no history of diabetes; and diabetes, defined as a fasting plasma glucose level of 7.0 mmol/L or greater (≥126 mg/dL) or a history of diabetes. Because subjects with lower glucose levels were expected to have a lower participation rate than those with higher glucose levels, we randomly selected participants from these groups according to a ratio of 1.44 (NGR) : 1.2 (IGR) : 1 (DM) to receive a more comprehensive survey including thyroid function test, a standard 75-g OGTT, blood and urine collection, an anthropometric measurement, and a questionnaire survey in phase 2 (June through August 2009).

A total of 3455 persons with blood and urine samples were included in phase 2 survey. Exclusion criteria were (i) participants having a personal history of overt hyperthyroidism, hypothyroidism, or thyroiditis and having been taking thyroxine or antithyroid drugs (*n* = 12); (ii) taking medications affecting thyroid function such as amiodarone, lithium, antipsychotic drugs, and antiepileptic drugs (*n* = 5); (iii) history of thyroidectomy (*n* = 2); (iv) participants with UACR ≥ 300 mg/g (*n* = 34). Moreover, 56 participants without complete thyroid function or UACR data were also excluded from the study. Finally, a total of 3346 subjects (1336 men and 2010 women) remained in the present analysis. The participants (3346 subjects) and the nonparticipants (6839 subjects) were similar in characteristics, such as sex and age distribution.

This study was approved by the Institutional Review Board of the Rui-Jin Hospital, Shanghai Jiao Tong University School of Medicine, and was in accordance with the principle of the Helsinki Declaration II. The written informed consent was obtained from each participant.

### 2.2. Clinical and Biochemical Measurements

Sociodemographics, medical history, and lifestyles including smoking and drinking status were conducted by trained personnel. Current smoking was defined as “yes” if a subject smoked at least one cigarette per day or seven cigarettes per week in the past 6 months. Current alcohol drinking was defined as “yes” if a subject consumed alcohol at least once a week in the past 6 months. Weight, height, and blood pressure were measured by experienced nurses. Body mass index (BMI) was calculated using the formula of weight/height^2^ (kilograms per squared meters). Three sitting blood pressure measurements taken consecutively at 1 min interval after at least 5 min rest using an automated electronic device (OMRON model HEM-752 FUZZY; Omron Co., Dalian, China) were averaged for analysis. Hypertension was defined when systolic blood pressure (SBP) was above 140 mmHg, when diastolic blood pressure (DBP) was above 90 mmHg, or when taking medication for blood pressure control.

All participants were admitted after an overnight fast of >10 h and two-point (0 and 2 h) OGTT with a 75 g glucose load was performed. Plasma and sera were obtained. The fasting and postloading venous blood samples were collected into a routine tube, respectively, and were immediately centrifuged on site at 4°C. Sera for the measurement of thyroid function were aliquot and kept frozen at −80°C until use. Blood glucose, serum creatinine, lipid profile including total cholesterol (TC), triglycerides, high-density lipoprotein cholesterol (HDL-C), low-density lipoprotein cholesterol (LDL-C), and high-sensitivity C-reactive protein (hs-CRP) were measured within 1 h of collection with an automated biochemical instrument (Bayer Biochemical autoanalyzer ADVIA 1650, Bayer, Leverkusen, Germany). Hyperlipidemia was defined according to the National Cholesterol Education Program Adults Treatment Panel III (NCEP ATPIII) criteria [13]. The abbreviated Modification of Diet in Renal Disease (MDRD) Study formula as recalibrated for Chinese was used to calculate the estimated glomerular filtration rate (eGFR) expressed in mL/min/1.73 m^2^ : 186 × (serum creatinine value in *μ*mol/L × 0.011) − 1.154 × (age) − 0.203 × (0.742 if female) × 1.233 (the number 1.233 is the adjusting coefficient for Chinese) [14].

A first-voided, early-morning spot urine sample was collected for assessing the UACR within 1 h after sample collection. All participants were advised to refrain from vigorous exercise before providing the urine sample. Urinary albumin concentration was measured using immunoturbidimetry (Beijia Biochemistry, Shanghai, China), and urinary creatinine concentration was determined by a modified Jaffe method on an automatic analyzer (Beckman LX/20, Brea, CA). UACR was calculated as urinary albumin concentration divided by creatinine concentration and expressed in mg/g. Microalbuminuria was defined as the UACR ranges within 30–300 mg/g and macroalbuminuria as ≥300 mg/g [2, 15].

### 2.3. Measurements of Thyroid Function

We measured thyroid stimulating hormone (TSH), free triiodothyronine (FT3), free thyroxine (FT4), thyroid peroxidase antibody (TPOAb), and thyroglobulin antibody (TgAb) levels. Thyroid function tests were analyzed in the Clinical Laboratory for Endocrinology, Shanghai Institution of Endocrine and Metabolic Diseases, which was certified by College of American Pathologists [16]. Serum FT3 (reference range: 2.62 to 6.49 pmol/L), FT4 (reference range: 9.01 to 19.04 pmol/L), and TSH (reference range: 0.35 to 4.94 *μ*IU/mL) were determined by chemiluminescent microparticle immunoassay method (Architect system; Abbott Laboratories, Abbott Park, IL). The coefficients of variations were 4.7–8.0% for FT3, 2.6–5.3% for FT4, and 3.1–3.4% for TSH. Serum anti-thyroid antibodies (TPOAb and TgAb) were measured by chemiluminescent microparticle immunoassay using Architect Anti-TPO and Anti-TG on the Architect i System (Abbott Laboratories). Reference ranges were TPOAb < 5.61 IU/mL (with an interassay CV of 4.3–6.8%) and TgAb < 4.11 IU/mL (with an interassay CV of 3.2–5.2%).

### 2.4. Statistics Analysis

All of the statistical analyses were performed using SAS version 9.2 (SAS Institute, Cary, NC), and a *P* value < 0.05 (two-sided) indicated statistical significance. Continuous variables were summarized as means ± SD or medians (interquartile ranges) for skewed variables. All categorical variables were presented as numbers and percentages. Triglycerides, fasting plasma glucose (FPG), OGTT 2-hour plasma glucose (OGTT-2 h PG), hs-CRP, FT3, FT4, TSH, TPOAb and TgAb, and UACR were normalized by logarithmically transformed before analyses because of nonnormal distribution.

The study participants were divided into four groups according to the quartiles of FT3. Demographic and metabolic features in each quartile were described and *P* for trend is measured using linear regression analysis across FT3 quartiles.

To investigate the association of FT3 categories and microalbuminuria, univariate- and multivariate-adjusted logistic regression models were performed with microalbuminuria as the dependent variable and FT3 categories as the independent variable along with the potential confounding factors. In the multivariable logistic regression models, we adjusted for the potential confounding factors including age, sex, BMI, smoking and drinking status, SBP, and DBP, TC, triglycerides, FPG and OGTT-2 h PG, TPOAb and TgAb, hs-CRP, use of angiotensin-converting enzyme inhibitor (ACEI) and/or angiotensin receptor blocker (ARB) drugs, and eGFR. Odds ratios (OR) with the lowest quartile were computed as the reference group.

To study if the association with FT3 and microalbuminuria was more prominent in some specific subgroups, we further performed stratified analyses on the associations between FT3 and the risk of microalbuminuria by the major risk factors including sex, age, BMI, hypertension, hyperlipidemia, diabetes according to OGTT diagnosis criteria, hs-CRP, and eGFR. Odds ratios were calculated for an increase of each 1 s.d. in log FT3 concentration in subgroups of the strata variables. Interactions of each above strata variable with the association of serum log FT3 and microalbuminuria risks were tested by introducing the variable × log FT3 into the univariate model.

## 3. Results

### 3.1. Characteristics of the Study Population

Among the 3,346 participants, the prevalence of microalbuminuria was 9.9% and no difference was found between males and females (9.2 versus 10.4%, *P* > 0.05). FT3 concentrations in male were significantly higher than that in female (*P* < 0.0001). Then participants were divided into four groups according to quartiles of FT3 with the first quartile representing the lowest one and the fourth quartile representing the highest one (≤4.28, 4.29–4.60, 4.61–4.93, and >4.94 pmol/L). The general characteristics of the four groups were presented in Table 1. Metabolic risk factors including sex distribution, current smoking or drinking status, BMI, DBP, triglycerides, TC, LDL-C, FT4, and eGFR increased significantly with the increment of FT3, whereas age, OGTT-2 h PG, HDL-C, TSH, and UACR decreased (all *P* for trend < 0.05). Compared with the participants in the lowest quartile of FT3, those in the second, the third, and the highest quartiles have significantly lower levels of UACR as follows: 6.08 (2.86–12.86) versus 5.93 (2.70–12.39), 5.93 (2.79–12.48), and 5.28 (2.65–12.12) mg/g (*P* for trend <0.05, Table 1). Nevertheless, no statistical difference was found for SBP, use of ACEI/ARB drugs, FPG, TgAb, TPOAb, and hs-CRP among the four groups.

### 3.2. Prevalence of Microalbuminuria in Different FT3 Levels

**Figure 1:**
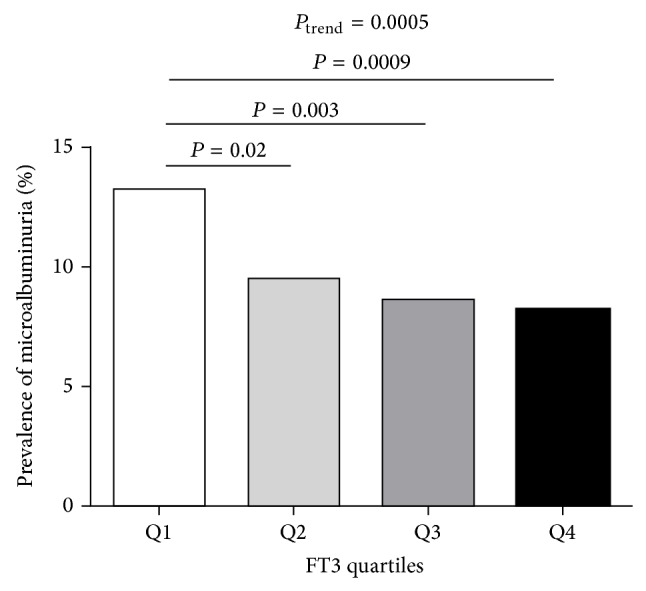
Prevalence of microalbuminuria in different FT3 quartiles: quartile 1 (Q1, *n* = 111), ≤4.28 pmol/L; quartile 2 (Q2, *n* = 80), 4.29–4.60 pmol/L; quartile 3 (Q3, *n* = 73), 4.61–4.93 pmol/L; quartile 4 (Q4, *n* = 67), >4.94 pmol/L.

The prevalence of microalbuminuria differed in the four groups with different FT3 quartiles. From the lowest quartile across to the highest one of FT3 quartiles, the prevalence of microalbuminuria was 13.2, 9.5, 8.6, and 8.2%, respectively (*P* for trend = 0.0005, Figure 1). Compared to the lowest FT3 quartile, the second, the third, and the highest quartiles showed a significant decrease in the prevalence of microalbuminuria (*P* = 0.02, 0.003, and 0.0009, resp.).

### 3.3. Association between FT3 and Microalbuminuria

In the univariable logistic regression model, the risk for microalbuminuria decreased across FT3 quartiles. The odds ratio (OR) for the highest FT3 quartile compared with the lowest quartile was 0.58, 95% confidence intervals (95% CI) 0.42–0.81, *P* = 0.001, and those in the second and third quartile were also less likely to have microalbuminuria (OR: 0.69, 95% CI, 0.51–0.93; *P* = 0.02 and OR: 0.62, 95% CI, 0.45–0.85; *P* = 0.003 resp.). The test for the trend was significant (*P* for trend = 0.0006). After adjusting for age, sex, BMI, and smoking and drinking status, participants in the second, third, and highest quartiles were still less likely to have microalbuminuria compared with those in the lowest quartile (adjusted OR: 0.72, 95% CI, 0.52–0.99, *P* = 0.04; 0.66, 95% CI, 0.48–0.91, *P* = 0.01; and 0.58, 95% CI, 0.41–0.82, *P* = 0.002, resp.). After further adjustments for other potential confounding factors including SBP, DBP, TC, triglycerides, FPG and OGTT-2 h PG, TPOAb and TgAb, hs-CRP, use of ACEI/ARB drugs, and eGFR, a fully adjusted logistic regression (Table 2) showed similar associations for the third and highest quartile but not for the second quartile (adjusted ORs for the highest versus the lowest quartile: 0.61, 95% CI, 0.43–0.87, *P* = 0.007, and for the third versus the lowest quartile: 0.70, 95% CI, 0.50–0.98, *P* = 0.04). The tests for the trend in the multivariable analyses were all significant (*P* for trend 0.01) (Table 2). We also conducted linear regression analyses for FT3 and UACR. In both simple and multivariate-adjusted linear regression analyses, FT3 levels were negatively and significantly associated with UACR (*P* values < 0.05).

Then we excluded the participants with abnormal FT3 levels (FT3 < 2.62 or ≥6.49 pmol/L, *n* = 41). The same analysis was performed for associations between FT3 and microalbuminuria. We found a similar inverse association between serum FT3 and microalbuminuria in unadjusted, adjusted, and fully adjusted model (Table 2). Compared with those in the lowest quartile, highest quartiles were still less likely to have microalbuminuria (OR 0.69; 95% CI, 0.49–0.98, *P* = 0.04) (Table 2). FT4 and TSH were not significantly associated with microalbuminuria in all the models generated (data not shown).

**Figure 2:**
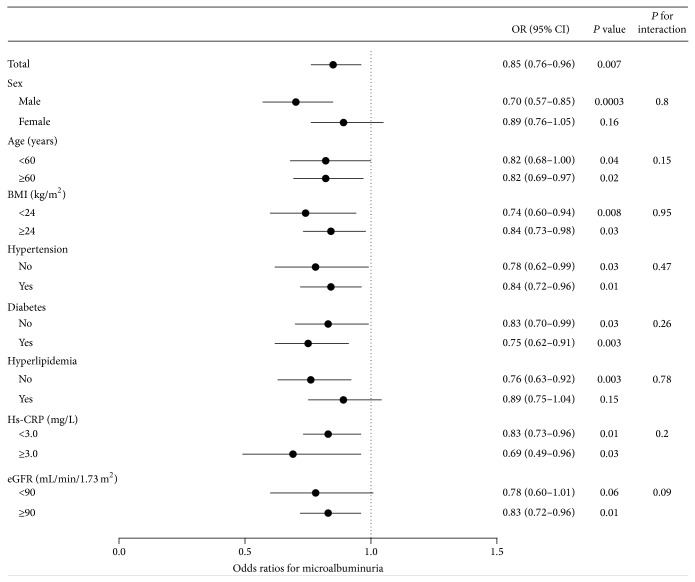
Adjusted odds ratios (OR) for each 1 s.d. increase in log FT3 concentration associated with the risk of microalbuminuria. Logistic regression model was adjusted for potential confounding factors including age, sex, BMI, smoking and drinking status, SBP and DBP, TC, triglycerides, FPG and OGTT-2 h PG, TPOAb and TgAb, hs-CRP, use of ACEI/ARB drugs, and eGFR (except for the strata variables). CI, confidence interval.

Furthermore, stratified analyses for multivariate-adjusted OR of microalbuminuria with each 1 s.d. increment in log FT3 concentration in different subgroups were conducted. The associations between microalbuminuria and FT3 were not consistently the same in subgroups. Inverse associations between microalbuminuria and FT3 were significant in both age strata (<60 and ≥60 years), both BMI strata (<24 and ≥24 kg/m^2^), both hypertension strata (yes and no), both diabetes strata according to the OGTT diagnosis criteria (yes and no), and both hs-CRP strata (<3 and ≥3 mg/L). Inverse associations between microalbuminuria and FT3 were also persisted in male, those without hyperlipidemia and eGFR ≥ 90 mL/min per 1.73 m^2^. No significant associations were detected in female, those with hyperlipidemia and eGFR < 90 mL/min per 1.73 m^2^ (Figure 2). Nevertheless, interactions between FT3 and these risk factors were not significant (*P* > 0.05) (Figure 2).

## 4. Discussion

In this community-based cross-sectional study, we found serum FT3 levels were inversely associated with microalbuminuria which is a powerful predictor of generalized vascular endothelial impairment. Moreover, the association was independent of other traditional vascular risk factors.

Microalbuminuria is a marker of increased risk of cardiovascular (CV) and renal morbidity and mortality in diabetic subjects. Meanwhile, microalbuminuria is a marker of increased risk for diabetes mellitus, hypertension, deterioration of renal function, and CV morbidity and all-cause mortality in nondiabetic subjects [5]. The prevalence of microalbuminuria is relatively high in previous studies. Jones et al. [17] reported the prevalence of microalbuminuria in 22,244 subjects aged from 6 to >80 years to be 7.8% (6.1% in males and 9.7% in females) with progressively increasing prevalence in adults > 40 years of age from the Third National Health and Nutritional Examination Survey (NHANES). Our data showed the prevalence of microalbuminuria was 9.9% (9.2% in males versus 10.4% in females) in participants aged 40 or above. Microalbuminuria is regarded as an integrated marker of systemic endothelial dysfunction and vascular disease via strong associations with blood pressure levels, metabolic status, lipids, and smoking habits [18]. In our study, we confirmed participants with microalbuminuria were more likely to have higher levels of BMI, SBP and DBP, FPG, OGTT-2 h PG, TC, triglycerides and hs-CRP (all *P* < 0.05), and lower levels of HDL-C (all *P* < 0.01) compared to those normoalbuminuria.

Thyroid hormone has crucial effects on the vascular system [19] and is also found to be associated with endothelial damage [20, 21]. Endothelial impairment has well-known effects on the development of hypertension, coronary artery disease, chronic heart failure, peripheral artery disease, diabetes, and chronic renal failure [22]. Serum FT3 level has been shown to be associated with impaired endothelial function as assessed by flow-mediated dilation in patients with chronic kidney disease [23]. By now no large-scale population-based studies have been published to focus on the association with thyroid function and microalbuminuria. Only one study [11] found an association between subclinical hypothyroidism and microalbuminuria in 147 prediabetic subjects. However, their sample size was small and the study was not population-based. In our study, after adjustment for a wide spectrum of lifestyle and biochemical risk factors we found FT3 was inversely associated with microalbuminuria in a large population. In addition, the association remained even in participants within normal FT3 level. These findings raised the possibility that normal thyroid function within the higher range was related to lower prevalence of microalbuminuria.

However, the exact mechanisms for inverse associations between FT3 and microalbuminuria are not well defined. Thyroid hormones have profound effects on vascular and endothelial functions [19–21]. Patients with subclinical hypothyroidism have endothelial dysfunction characterized by reduced endothelium-dependent vasodilatation and impaired nitric oxide availability. Moreover, this abnormal vascular profile is partially independent of dyslipidemia and restored by levothyroxine treatment [7, 24]. Vascular function is impaired in patients with mild and subclinical hypothyroidism, as documented by the increase in systemic vascular resistance and arterial stiffness and by the impaired endothelial function. The previous study showed the T3-induced decrease in systemic vascular resistance may be explained by direct modulation of endothelium-dependent and -independent vasoregulation [9]. In experimental models, it was also shown that FT3 had antiatherosclerotic effects on the vascular bed via its effect on mitochondrial oxidation systems, induction of vasodilatatory molecules, and inhibition of angiotensin II receptor expression and downstream signal transduction [8, 25]. Thus, endothelial damage/dysfunction, closely associated with the pathogenesis of atherosclerosis [26], impairs endothelium-dependent vasodilatation [27] which may mediate with microalbuminuria. It is highly possible that low FT3 could be a marker of endothelial dysfunction similar to presence of microalbuminuria and both might be mediated by unmeasured or unknown factor.

In addition, thyroid hormone is closely associated with the metabolic syndrome (Mets), a syndrome of insulin resistance, obesity, hypertension, and dislipidemia. The data from NHANES III showed strong positive associations between microalbuminuria and Mets [28]. Meanwhile, insulin resistance is generally hypothesized to be the major underlying pathophysiological process of Mets [29]. Low thyroid function (even in the euthyroid state) was reported to be associated with increased insulin resistance [30], which might mediate the association with microalbuminuria [31].

Microalbuminuria has been generally regarded as a marker of generalized vascular endothelial impairment [32]. Xiang et al. [33] reported endothelial dysfunction exists in euthyroidism patients with Hashimoto's disease and autoimmune reactivity, indicating TPOAb might be responsible for the endothelial dysfunction and the subsequent microalbuminuria. However, our data showed the link with microalbuminuria was roughly similar for FT3 with and without TPOAb. This demonstrates that the effect of FT3 on microalbuminuria might be independent of thyroid autoimmunity. Additionally, increasing evidence shows that systemic and vascular low-grade inflammation, as reflected by increased serum concentration of hs-CRP, may provide a mechanism for the pathogenesis of microalbuminuria [34]. However, we found FT3 inversely associated with microalbuminuria either in subjects with hs-CRP ≥ 3.0 mg/L or <3.0 mg/L, indicating that the association between FT3 and microalbuminuria cannot be explained by systemic inflammation.

Our study was the first to report an independent association between FT3 concentration and microalbuminuria in a large population. Our study provided confirmatory evidence for the association between FT3 and microalbuminuria. However, limitations are unavoidable. Firstly, our study was cross-sectional designed, and no causal relationships can be established. Further longitudinal research is required to clarify the cause and effect relationships. Secondly, the urinary albumin excretion was measured on a morning spot-urine sample. We acknowledge that a 24 h urine sample would provide more stable measures for urinary albumin excretion. However, it was reported that the use of spot specimens for urinary albumin-to-creatinine ratio was more convenient for a large cohort survey and the results correlated well with 24 h urinary albumin excretion and could be used as a reliable alternative to 24 h urinary albumin excretion in large epidemiological research [35]. Thirdly, our study included only middle-aged and elderly subjects, and the results may not be applied to a general population with different age composition.

## 5. Conclusions

In summary, the present study shows inverse correlation between microalbuminuria, an established marker of generalized vascular endothelial impairment, and serum FT3. FT3 concentrations may play a role in the pathogenesis of microalbuminuria. Moreover, this inverse association also exists in subjects classified as being euthyroid, which implicates low normal thyroid function could adversely affect microalbuminuria even in euthyroidism. We therefore propose to consider FT3 levels in addition to Mets and other risk factors that are traditionally associated with microalbuminuria [5]. Further studies are needed to explore the mechanisms responsible for the correlation between FT3 and microalbuminuria.

## Figures and Tables

**Table 1:** General characteristics of the study population.

	FT3 quartiles (pmol/L)				
	Quartile 1	Quartile 2	Quartile 3	Quartile 4	*P* for trend
	(*n* = 839)	(*n* = 843)	(*n* = 845)	(*n* = 819)	
Male	275 (32.8)	293 (34.8)	329 (38.9)	439 (53.6)	<0.0001
Age (years)	63.6 ± 11.1	61.0 ± 10.1	60.1 ± 9.5	58.4 ± 8.3	<0.0001
BMI (kg/m^2^)	24.9 ± 3.7	25.3 ± 3.7	25.3 ± 3.8	25.7 ± 3.5	<0.0001
Current smokers	120 (14.3)	154 (18.3)	163 (19.3)	251 (30.7)	<0.0001
Current drinkers	111 (13.2)	125 (14.8)	129 (15.3)	160 (19.5)	0.0006
Use of ACEI/ARB drugs	33 (3.93)	32 (3.80)	31 (3.67)	33 (4.03)	0.98
SBP (mmHg)	139.5 ± 23.5	137.6 ± 22.2	138.6 ± 21.2	138.9 ± 20.4	0.77
DBP (mmHg)	76.3 ± 10.1	77.8 ± 10.2	78.6 ± 9.6	81.0 ± 10.5	<0.0001
FPG (mmol/L)	5.2 (4.7–6.1)	5.2 (4.8–6.1)	5.2 (4.8–5.8)	5.3 (4.8–6.0)	0.08
OGTT-2 h PG (mmol/L)	8.5 (6.3–14.4)	7.8 (6.1–12.5)	7.6 (6.1–11.4)	7.7 (6.3–11.4)	<0.0001
Triglycerides (mmol/L)	1.28 (0.89–1.89)	1.40 (1.00–2.02)	1.46 (1.01–2.14)	1.61 (1.10–2.34)	<0.0001
TC (mmol/L)	5.07 ± 1.04	5.15 ± 0.98	5.21 ± 0.93	5.17 ± 0.99	0.02
HDL-C (mmol/L)	1.37 ± 0.33	1.36 ± 0.29	1.37 ± 0.32	1.32 ± 0.29	0.01
LDL-C (mmol/L)	2.34 ± 0.71	2.41 ± 0.70	2.39 ± 0.66	2.42 ± 0.68	0.03
FT3 (pmol/L)	4.05 (3.84–4.18)	4.45 (4.37–4.53)	4.76 (4.68–4.85)	5.18 (5.05–5.38)	—
FT4 (pmol/L)	13.7 (12.7–14.9)	14.1 (13.2–15.1)	14.2 (13.3–15.3)	14.8 (13.8–15.8)	<0.0001
TSH (*μ*IU/mL)	1.59 (1.07–2.33)	1.50 (1.02–2.18)	1.44 (1.01–2.160	1.35 (0.92–1.99)	<0.0001
TPOAb (IU/mL)	0.30 (0.17–0.74)	0.31 (0.17–0.77)	0.30 (0.16–0.72)	0.34 (0.18–0.81)	0.93
TgAb (IU/mL)	1.09 (0.70–2.73)	1.08 (0.70–2.95)	1.02 (0.71–2.27)	1.05 (0.72–2.25)	0.08
eGFR (mL/min/1.73 m^2^)	107.5 ± 28.6	112.6 ± 24.4	112.8 ± 24.9	115.8 ± 22.9	<0.0001
UACR (mg/g)	6.08 (2.86–12.86)	5.93 (2.70–12.39)	5.93 (2.79–12.48)	5.28 (2.65–12.12)	0.03
Hs-CRP (mg/g)	0.21 (0.05–1.13)	0.18 (0.06–0.85)	0.18 (0.07–0.61)	0.24 (0.08–0.82)	0.58

Data were means ± SD or medians (interquartile range) or numbers (proportions). BMI, body mass index; ACEI, angiotensin-converting enzyme inhibitor; ARB, angiotensin receptor blocker; SBP, systolic blood pressure; DBP, diastolic blood pressure; TC, total cholesterol; HDL-C, high-density lipoprotein cholesterol; LDL-C, low-density lipoprotein; FPG, fasting plasma glucose; OGTT-2 h PG, OGTT 2-hour plasma glucose; FT3, free triiodothyronine; FT4, free thyroxine; TSH, thyroid stimulating hormone; TPOAb, thyroid peroxidase antibody; TgAb, thyroglobulin antibody; eGFR, estimate glomerular filtration rate; UACR, urinary albumin-to-creatinine ratio.

FT3 quartiles were as follows: quartile 1, ≤4.28 pmol/L; quartile 2, 4.29–4.60 pmol/L; quartile 3, 4.61–4.93 pmol/L; quartile 4, >4.94 pmol/L.

**Table 2:** The risk of microalbuminuria according to quartiles of FT3.

	FT3 quartiles (pmol/L)				
	Quartile 1	Quartile 2	Quartile 3	Quartile 4	*P* for trend
In total participants (*n* = 3346)					
Cases/participants	111/839	80/843	73/845	67/819	
Model 1	1.00	0.69 (0.51–0.93)	0.62 (0.45–0.85)	0.58 (0.42–0.81)	0.0006
Model 2	1.00	0.72 (0.52–0.99)	0.66 (0.48–0.91)	0.58 (0.41–0.82)	0.005
Model 3	1.00	0.74 (0.53–1.02)	0.70 (0.50–0.98)	0.61 (0.43–0.87)	0.007
In participants with normal FT3 levels (*n* = 3305)					
Cases/participants	111/831	79/841	65/822	73/811	
Model 1	1.00	0.67 (0.50–0.91)	0.56 (0.40–0.77)	0.64 (0.47–0.88)	0.002
Model 2	1.00	0.71 (0.51–0.97)	0.60 (0.43–0.84)	0.65 (0.46–0.91)	0.02
Model 3	1.00	0.73 (0.52–1.01)	0.64 (0.45–0.91)	0.69 (0.49–0.98)	0.02

See Table 1 for the FT3 quartiles definition. Data are odds ratios (ORs, 95% confidential interval).

Model 1 is unadjusted.

Model 2 is adjusted for age, sex, BMI, and smoking and drinking status.

Model 3 is adjusted for age, sex, BMI, smoking and drinking status, SBP and DBP, TC, triglycerides, FPG and OGTT-2 h PG, TPOAb and TgAb, hs-CRP, use of ACEI/ARB drugs, and eGFR.
